# Effects of maternal depression on maternal responsiveness and infants’ expressive language abilities

**DOI:** 10.1371/journal.pone.0277762

**Published:** 2023-01-11

**Authors:** Ruth Brookman, Marina Kalashnikova, Penny Levickis, Janet Conti, Nan Xu Rattanasone, Kerry-Ann Grant, Katherine Demuth, Denis Burnham

**Affiliations:** 1 The MARCS Institute for Brain, Behaviour and Development, Western Sydney University, Penrith, NSW, Australia; 2 School of Psychology, Western Sydney University, Penrith, NSW, Australia; 3 BCBL, Basque Center for Cognition, Brain and Language, San Sebastian, Spain; 4 IKERBASQUE, Basque Foundation for Science, Bilbao, Spain; 5 Graduate School of Education, University of Melbourne, Melbourne, Victoria, Australia; 6 Child Language Lab, Centre for Language Studies, Department of Linguistics, Macquarie University, Macquarie Park, NSW, Australia; 7 Health Education and Training Institute, Parramatta BC, NSW, Australia; University of Zurich, SWITZERLAND

## Abstract

High levels of maternal responsiveness are associated with healthy cognitive and emotional development in infants. However, depression and anxiety can negatively impact individual mothers’ responsiveness levels and infants’ expressive language abilities. Australian mother-infant dyads (N = 48) participated in a longitudinal study examining the effect of maternal responsiveness (when infants were 9- and 12-months), and maternal depression and anxiety symptoms on infant vocabulary size at 18-months. Global maternal responsiveness ratings were stronger predictors of infants’ vocabulary size than levels of depression and anxiety symptoms. However, depression levels moderated the effect of maternal responsiveness on vocabulary size. These results highlight the importance of screening for maternal responsiveness–in addition to depression–to identify infants who may be at developmental risk. Also, mothers with elevated depression need support to first reduce their symptoms so that improvements in their responsiveness have the potential to be protective for their infant’s language acquisition.

## Introduction

There is a strong link between parenting behaviours and children’s language abilities (see [1] for a meta-analysis). Maternal responsiveness is one measure of parenting behaviour, and it refers to the quality of maternal contributions in interactions with their infants [2]. It is the keystone maternal component in a three-stage sequence of communication: child’s action–maternal reaction–effect on the child [3]. Maternal responsiveness has been defined as maternal behaviours that are contingent, follow rather than re-direct, and build on the infant’s focus of attention and activity [4]. The inclusion of ‘contingency’ in any definition of responsiveness is important as it highlights that responsiveness occurs in the context of a reciprocal process. While dyadic in nature, it is generally considered to be the mother’s responsibility to establish and monitor attunement within the mother–infant interaction [5]. Mothers who are highly responsive are sensitive to their infants’ cues and respond to them reasonably quickly while establishing clear contingency and in ways that are well matched to their infants’ developmental level [6]. In doing so, they facilitate their infants’ secure attachment, self-efficacy, motivation, and language skills [7–10].

Individual differences in maternal responsiveness have been linked to mothers’ emotional health concerns such as depression and anxiety symptoms (e.g., [11–14]). However, this relation does not appear to be linear since it is not always the case that mothers who experience symptoms of depression and anxiety also develop low levels of maternal responsiveness in interactions with their infants [15]. Previous research has indicated that infants whose mothers experience depression or anxiety exhibit delays in different aspects of language development, but it is not clear whether these language outcomes are linked to maternal mental health symptoms, to individual differences in maternal responsiveness, or the interaction of these two factors. The present longitudinal study investigated this complex relation by assessing the effects of maternal depression and anxiety symptoms and maternal responsiveness levels during their infant’s first year of life on infants’ developing vocabulary skills at 18-months of age.

### Maternal responsiveness and links with infant language development

Maternal responsiveness is nested within the social interactive dimension of early language development, highlighting the bi-directionality in the interaction between a child’s neurobiological makeup and their environment [16, 17]. In addition to endogenous biological factors, early language development is proposed to be facilitated by a cumulative process of reciprocal social interactions between infants and their caregivers [18]. As caregivers support infants’ exploration and engagement with their environment, caregivers’ responsiveness operates through a process of reciprocal adjustments that impact child language, cognitive development, and attachment [19, 20].

Maternal responsiveness is proposed to contribute positively to infants’ rapidly emerging language abilities from the first months of life [10, 21]. Maternal responses to infants’ babbling promote infants’ communication skills [22]. For example, infants’ vocalisations are longer and more complex following maternal responses (e.g., vocalisations, smiles, and gestures) that are contingent to infants’ communication cues (see [23–25]). As children approach their first birthdays and develop more sophisticated linguistic abilities, mothers’ sensitive responding continues to scaffold infants’ emerging language abilities. There is evidence that maternal responsiveness predicts infant concurrent and future vocabulary size [26, 27] and the timing of expressive language milestones [28]. Importantly, Tamis-LeMonda et al. [28] found that maternal responsiveness–as coded from video recordings of mother–infant interactions when the infants were 9- and 13-months–predicts five language-acquisition milestones: first imitations, first spontaneous words, achievement of 50-word expressive vocabulary size, combinatorial speech, and the ability to use language to refer to the past.

### Maternal responsiveness and emotional health concerns

Maternal responsiveness and children’s language outcomes can be negatively impacted by the presence of maternal depression and anxiety symptoms [11–14]. Prevalence data suggests that women are most at risk of being diagnosed with depression during their child-bearing years, and that up to one-third of women will experience at least one major depressive during their lifetime [29]. During the postnatal period, depression is commonly referred to as postnatal depression (PND), with symptoms corresponding to major depressive disorder (MDD) [33]. Postnatal depression symptoms can reduce women’s quality of life and their ability to engage with their baby. Symptoms can include a loss of appetite, feelings of unworthiness and guilt, low motivation, and loss of interest in activities that were previously enjoyable [30]. Anxiety symptoms also commonly co-occur with depression during the postnatal period [14, 31]. Women who experience excessive worry and anxiety may also experience significant levels of distress that can impact negatively on their daily functioning and ability to care for their infant [32]. Elevated anxiety symptoms can be associated with a formal diagnosis of generalised anxiety disorder, panic disorder, and specific phobias [33].

Research evidence has established a relationship between maternal depression symptoms and the language development of very young children. For example, one large longitudinal study examined mothers and their infants from 6-months through to 3-years of age [34]. Results demonstrated that children obtained lower scores at aged 3-years on expressive language measures when their mothers had elevated depression scores. Correspondingly, a cross sectional study with mother–infant dyads found a negative correlation between the severity of maternal depression scores and infants’ expressive language scores at 12-months of age [35]. More recently, Brookman et al. [36] assessed infants’ lexical abilities at 18-months of age and found that infants of mothers with elevated depression and anxiety symptoms showed poorer performance in a familiar word recognition task compared to controls. Furthermore, these effects were not restricted to mother-infant dyads in which the mother had elevated depression symptoms. When maternal depression scores were treated as a continuum to include mothers with sub-clinical depression levels, these continued to be negatively correlated with infants’ lexical processing efficiency and vocabulary size. Empirical research examining maternal anxiety and infant language development is comparatively scarce and findings are less consistent than studies examining maternal depression [37, 38]. However, pre-and-postnatal anxiety has also been adversely linked with child developmental outcomes (see [39, 40]), including with infant expressive language abilities at 12-months of age [41].

According to the evidence reviewed above, maternal depression and anxiety symptoms may yield direct adverse effects on infants’ language development. Another possibility is that these effects are indirect and driven by the negative relation between depression and anxiety and maternal responsiveness [15]. Depression and anxiety symptoms can disrupt a mother’s maternal responsiveness levels, whereby she has difficulty recognising her infant’s communication cues and responding to them in an age-appropriate, prompt and contingent manner [42, 43]. In fact, maternal depression has been the most commonly cited factor that adversely impacts maternal responsiveness [34, 44]. Mothers with depressed mood have been observed to interact with their infants with either an intrusive (over-stimulating) communication style or a withdrawn [under-stimulating] communication style [45]. A large-scale longitudinal study followed over 1000 children from 4-weeks of age through to school entry where the trajectory of maternal depression symptoms were assessed together with maternal responsiveness levels [13]. Children’s cognitive ability and social skills were also assessed. Findings yielded a negative association with the severity of maternal depression symptoms, the level of maternal responsiveness, and child outcomes.

The relation between mother-infant interactions and maternal anxiety are studied less often than depression; however, disruptions have been observed within anxious mother–infant dyads [12]. For example, there is evidence that mothers with high anxiety are less engaged with their infants and also less responsive [14, 46]. Also, a study examining maternal interactions with 3-month-old infants, found that when mothers with depression also had higher levels of state anxiety, their responses were more intrusive and less sensitive when compared with a control group [47].

Previous research has not directly investigated the extent to which depression and anxiety moderate the relation between maternal responsiveness and infants’ language outcomes, which was the main objective of this study. However, a recent study by Brookman and colleagues [48] provides evidence that this may be the case by showing that maternal emotional health symptoms moderate the relation between measures of infants’ home language environment and their vocabulary size. The study involved a group comparison of the home language environment of infants with mothers affected by emotional health concerns versus a control group. Findings from day-long audio recordings obtained during infants’ first year of life (6- and 12-months) revealed the infants who had mothers with emotional health concerns, vocalised less than controls. These infants were also exposed to fewer conversational turn counts across the day. This individual variability in early home language environment was a significant predictor of infants’ expressive vocabulary size in the second year of life (18-months) over-and-above maternal emotional health symptoms. Importantly, maternal anxiety was shown to have a moderation relation with home language measures and infant vocabulary. These findings suggest that depression and anxiety symptoms may moderate the effects of maternal responsiveness on children’s cognitive outcomes.

### Measuring maternal responsiveness

Empirical research has employed various methods for assessing maternal responsiveness, such as observer-based and self-report survey-based approaches. Observer-based approaches typically involve video recordings of maternal interactions with their infant during activities such as playtime [49] and joint book reading [50]. These recordings are rated either in real-time or video recorded for offline ratings. *Detailed* observer-based measures provide fine-grained information about maternal interactions with their infant, but they can be time-consuming, thus limiting their utility in clinical settings. Alternatively, *global* measures provide either a single measure designed to capture all the critical elements of maternal responsiveness in one score [51, 52] or separate ratings of specific maternal behaviours [53]. Global rating scales provide a cost-effective alternative to more detailed measures and have been applied in both research and clinical settings [54, 55].

A recent longitudinal study employed a global rating scale to examine the association between maternal responsiveness and language abilities in a community-based sample of ‘slow-to-talk’ toddlers [16]. At 2-years of age, a five‐point global rating scale (1 = very low; 5 = very high) was used to rate maternal responsiveness during a 15‐minute play session between mothers and their toddlers. A follow-up language assessment of the children (ages 3- and 4-years) showed that mother’s earlier maternal responsiveness scores (2-years) strongly predicted their children’s later receptive and expressive language skills. To extend these findings, the present study examined the continuity of global measures taken during the first year of life, by comparing maternal responsiveness scores in assessments with infants at 9- and 12-months of age.

### The present study

The overall aim of this study was to examine the relations between maternal responsiveness assessed in the infants’ first year of life, maternal depression and anxiety symptoms, and children’s language abilities in their second year. Understanding the interaction between these factors has the potential to lead to significant practical implications as this research can provide evidence that in addition to treating maternal depression and anxiety symptoms, assessment and enhancement of maternal responsiveness could mitigate developmental risks for infants of mothers with emotional health concerns.

The sample consisted of mother–infant dyads who were a subset of the longitudinal cohort from Brookman and colleagues [48]. Maternal responsiveness was assessed in maternal interactions when infants were 9- and 12-months old. An observer-based global rating scale of maternal responsiveness was employed. Global rating scales have the advantage of providing ratings of maternal responsive behaviours with high predictive validity compared to survey-based approaches [56, 57]. In addition, self-reported measures were used to assess the severity of maternal depression and anxiety symptoms. The severity of maternal depression and anxiety symptoms were treated as continuous variables in our design since elevated symptoms that do not reach clinical levels have also been associated with considerable functional and social impairments in mothers [57]. Maternal mental health measures were collected at four time points (when infants were 6-, 9-, 12-, and 18-months) and averaged for analyses. This approach was chosen since it accounts for variability in the onset and duration of symptoms during the postnatal period from birth to 18-months, which was of interest for this study. A parental report [58] was used to obtain a measure of infants’ expressive vocabulary size at when they were 18-months of age. This is the developmental age typically associated with a landscape change in infants’ expressive vocabulary skills and commonly referred to as the “vocabulary spurt” [59–62].

Two research objectives were pursued in this study. First, we investigated whether maternal responsiveness in the first year of an infant’s life is linked with infant vocabulary size at 18-months. Second, we investigated the direct and indirect relations between maternal depression and anxiety scores, levels of maternal responsiveness, and infant vocabulary size. we hypothesised that maternal responsiveness ratings in the first year of life would significantly correlate with infants’ expressive vocabulary scores at 18-months [28]. In regard to the second objective, we hypothesised that maternal responsiveness scores would predict expressive vocabulary size at 18-months. This was also the case for maternal depression and anxiety scores, which were also predicted to relate to infants’ vocabulary. Critically, we hypothesised that maternal responsiveness would predict variance in infants’ vocabulary scores over and above the variance explained by maternal depression and anxiety scores in our sample. In addition, we used a moderation analysis to assess the indirect role of depression and anxiety as moderators of the relation between maternal responsiveness and infants’ vocabulary [48].

## Materials and method

### Participants

The participants in this study were forty-eight mother–infant dyads (25 female and 23 male infants). The mothers were aged between 25 to 40 years (*M* = 32.90 years, *SD* = 3.90). Socio-economic status was indexed by maternal education levels that ranged from high school to postgraduate degree (Median = university degree). All of the infants recorded a birth weight within the normal range. They were born full-term (37–42 weeks) and into monolingual households with both parents present. Infants had no reported hearing loss, significant health problems or neurological difficulties (see Table 1 for detailed demographic information of the sample).

**Table 1:** Demographic information about the mother–infant dyads.

Characteristic	Infant age			
	Birth	6 months	12 months	18 months
Maternal Age (years)				
Range	25–40			
Mean (SD)	32.90 (3.90)			
Maternal Education: *n* (%)				
1. High School	2 (4)			
2. Diploma or trade	6 (12)			
3. Undergraduate degree	27 (56)			
4. Postgraduate degree	13 (28)			
Infant gender: *n* (%)				
Male	23 (48)			
Female	25 (52)			
Birth weight (kg)				
Range	2.38–4.89			
Mean (SD)	3.50 (.52)			
Birth order: *n* (%)				
First-born	17 (35)			
Maternal Paid employment: *n* (%)				
Nil paid work		20 (42)	13 (27)	10 (21)
Part-time paid work		23 (48)	29 (61)	30 (63)
Full-time paid work		5 (10)	6 (12)	8 (16)
Hours paid childcare/week: *n* (%)				
Less than 10		43 (90)	21(44)	12 (25)
10–20		2 (4)	15 (31)	18 (38)
20–30		2 (4)	6 (13)	10 (21)
30–40		1 (2)	4 (8)	6 (12)
More than 40		0	2 (4)	2 (4)

There were two main recruitment methods for this study. First, fourteen mother-infant dyads were recruited from a community sample of mothers previously enrolled in a large-scale longitudinal project examining maternal anxiety in the prenatal period and infants’ cortisol levels at 12-weeks of age. Second, the remaining mother–infant dyads were recruited from an infant laboratory database and study flyers distributed on social media platforms and through community agencies, playgroups, and libraries. The sub-samples recruited using the two strategies did not differ significantly in infant gender, maternal age, and maternal education. Informed written consent was obtained from each mother for research participation for themselves and their infant. This study was approved by the Western Sydney University Human Research Ethics Committee (approval number: H11703).

### Maternal depression and anxiety

Mothers’ depression symptoms were examined via the Centre for Epidemiologic Studies Depression Scale-Revised (CESD-R) [63]. The CESD-R is a 20-item self-reported scale that measures symptoms of depression. All items are measured on a 5-point Likert scale (0 = “not at all” to 4 = “nearly every day for the last 2 weeks”). Examples of items include: “My appetite was poor” and “I did not like myself”. A total score of ≥ 16 indicates clinical levels of depression. The CESD-R has been previously used in perinatal populations [64], [65], and has been demonstrated to have excellent psychometric properties [66].

Mothers’ anxiety symptoms were examined using the State Scale of the State-Trait Anxiety Inventory (STAI) [67]. The State subscale of the STAI is a self-reported measure of current (‘in the moment’) anxiety symptoms. All 20 items are measured on a 4-point forced-choice scale (1 = ‘not at all’ to 4 = ‘very much’). Examples of items include: “I am tense; I am worried” and “I feel calm; I feel secure.” The State subscale of the STAI is commonly used to measure state anxiety levels in women during the perinatal period where a score ≥ 40 indicates clinically high levels of state anxiety [39, 67, 68]. The STAI has demonstrated good psychometric properties with both clinical and non-clinical populations [67].

Mothers completed these measures at four different time points during the postnatal period (6-, 9-, 12- and 18-months). Correlations among individual scores across timepoints ranged from moderate to high (.362 - .748 for CESD-R; .429 - .791 for STAI), confirming the reliability and stability of these assessments in our sample (see S1 Table). However, these correlations also showed that as expected, there was some variation in the severity and persistence of maternal depression, which is known to influence child development outcomes [34]. To account for this variation, scores were averaged across the data collection points to calculate a mean postnatal depression and anxiety score for each participant (see Table 2).

**Table 2:** Descriptive statistics for maternal depression and anxiety, maternal responsiveness, and infant expressive vocabulary measures.

	Infant age				Postnatal Mean Score
	6 months	9 months	12 months	18 months	
Depression	*n* = 38	*n =* 43	*n =* 45	*n =* 43	*n =* 48
Range	0–28	0–23	0–27	0–26	0–18
Mean (SD)	7.21 (6.23)	7.33 (6.79)	6.87 (6.21)	6.79 (6.32)	6.65 (5.29)
Percentage	5	12	9	9	
Anxiety	*n* = 38	*n* = 43	*n* = 45	*n* = 43	*n =* 48
Range	21–47	20–55	20–51	20–62	21–45
Mean (SD)	31.18 (7.46)	32.86 (8.74)	31.87 (8.02)	31.19 (9.85)	31.30 (7.08)
Percentage	11	19	18	16	
Maternal Responsiveness		*n =* 39	*n =* 46		*n =* 48
Range		1–4	2–5		1.5–4
Mean (SD)		2.92 (.88)	3.09 (.87)		2.98 (.84)
Vocabulary size				*n =* 46	
Range				7–272	
Mean (SD)				68.77 (56.58)	

Note. Depression = CESD-R score; Anxiety = STAI score; Maternal responsiveness = PaRRiS score; Vocabulary size = OZI expressive vocabulary score. Percentage = the proportion of mothers with scores at or above the clinical threshold.

### Maternal responsiveness

Mothers and their infants were invited to participate in a brief play session in a child-friendly laboratory room when the infants were 9- and 12-months old. Some mothers were unable to attend one of the laboratory visits at either the 12-month (*n* = 2) or 9-month visit (*n* = 9). Single recordings for these dyads were still included in the analysis. Mothers were asked to interact with their infants using a set of age-appropriate toys including a toy phone, a four-piece jigsaw puzzle, a doll, nesting cups, two matching shakers/rattles, a roller toy with a mirror and movable parts, and a book. The mothers were informed that the play session would be video-recorded and were asked to interact with their baby as they normally would at home. The mother and infant sat on the floor during the interaction. The experimenter [first author] sat in the same room and recorded the interaction using a tablet, which increased the flexibility to move with the infant to different locations in the room if required. The researcher remained silent and did not engage in the interaction in any way. The duration of the recording was approximately five to 10 minutes.

#### The Parental Responsiveness Rating Scale [PaRRiS]

The PaRRiS measurement tool was used to score the quality of the mother–infant interactions during the 5- to 10-minute play session. The PaRRiS was adapted from the Marfo global rating scale of responsiveness [69]. The videos were rated in real time, without rewinding or pausing the recording. A score was then assigned for overall maternal responsiveness using the PaRRiS five-point Likert-style scale (1 = Very Low; 5 = Very High responsiveness) [55]. Details of the rating scale can be found in S1 File.

A total of 85 mother–infant play sessions were obtained (39 at age 9-months; 46 at age 12-months) and rated for maternal responsiveness. The experimenter (first author) was trained in the use of the PaRRiS by the third author and co-developer of the scale. The third author, who was unaware of participants’ maternal depression and anxiety scores, provided blind ratings for 32 (38%) of the videos. Coding and reliability checks occurred intermittently during the study to ensure that there was no change in inter-rater consistency over time. The occasional difference between raters’ scores did not exceed one-point. These were then discussed until arriving to a consensus before they were applied to each participant’s video. Overall, the two coders achieved a high level of reliability (Cohen’s *Kappa* = .853, *p* < .001).

### Expressive vocabulary size

When the infants were 18-months of age, mothers completed the Australian English adaptation [70] of the MacArthur-Bates Communicative Inventory [71]. The OZI is a vocabulary checklist consisting of 558 words that are likely to be familiar to infants from 12- to 30-months. The checklist was completed by mothers who were asked to identify words that they have heard their infant produce. Data for one infant was not included due to failure to complete the checklist. Two participants’ OZI scores were classified as outliers and excluded from further analysis because they were three standard deviations above and below the mean respectively [72].

## Results

### Maternal responsiveness

Descriptive statistics for all measures are presented in Table 2. First, preliminary analyses were conducted to evaluate the stability of Maternal Responsiveness scores obtained using the global rating scale when infants were 9- and 12-months of age. A Wilcoxon signed ranks test comparing individual Maternal Responsiveness scores at 9-months (*M* = 2.92, *SD* = .88) and 12-months (*M* = 3.09, *SD* = .87) showed that 12-month scores were significantly higher than 9-month scores (*Z* = 2.50, *p* = .012). A follow up correlation analysis revealed that the 9- and 12-month maternal responsiveness scores were strongly correlated, *r* (34) = .75, *p* < .001. Due to the high correlation between the two measures, maternal responsiveness ratings at 9- and 12-months were averaged to provide a mean maternal responsiveness score for the first year of life, which was used in all subsequent analyses. For those dyads who attended mother-infant play sessions at only one time-point (*n* = 11), this score was the same as the average score.

### Relation between maternal responsiveness, depression and anxiety scores, and infants’ vocabulary size

First, to assess the relations between maternal responsiveness, depression and anxiety scores, and infants’ future vocabulary size, Pearson correlations were conducted. Infants’ expressive vocabulary size was negatively correlated with their mothers’ mean anxiety scores (*r*(45) = -.36, *p* = .014) and the correlation with mean depression scores approached significance (*r*(45) = -.28, *p* = .068). On the contrary, infants’ vocabulary size was positively correlated with maternal responsiveness ratings (*r*(48) = .37, *p* = .013). Interestingly, mean maternal responsiveness scores were not significantly correlated with mean depression (*r*(45) = .019, *p* = .902) or anxiety scores (*r*(45) = -.256, *p* = .09). Additional correlation analyses included maternal age and education. Maternal age was significantly negatively correlated to maternal responsiveness but not to any other maternal and infant measure, and maternal education was not significantly correlated with any maternal or infant measure in this study (see S2 Table).

Second, to assess the individual contribution of each maternal factor to infants’ vocabulary size scores, we conducted a hierarchical multiple regression analysis. The model included infant expressive vocabulary size as the dependent variable. Maternal depression and anxiety scores were entered as predictor variables in Step 1, and maternal responsiveness ratings were entered in Step 2. This step order was determined by our hypothesis that after accounting for maternal depression and anxiety scores, maternal responsiveness should explain a significant amount of variance in infant vocabulary scores at 18-months. The resulting model is presented in Table 3. The model explained 16% of variance, and maternal responsiveness was the only significant predictor of infant vocabulary scores. Given the significant correlation between maternal age and maternal responsiveness levels in our sample and previous evidence that maternal education is a significant predictor of infant language outcomes (e.g., [73]), these two factors were also included in a preliminary model, but they did not yield significant effects (see S3 Table).

**Table 3:** Multiple regression model with maternal emotional health and maternal responsiveness as the predictor variables and expressive vocabulary size as the dependent variable.

	**Step 1, *Adj R*^*2*^ = .92, *F*(2, 42) = 3.22, *p* = .050**			
**Predictors**	***β***	***SEM***	***t***	***p***
Mean postnatal depression	-.042	2.137	-.208	.836
Mean postnatal anxiety	-.334	1.608	-1.667	.103
	**Step 2, *Adj R*** ^***2***^ **= .163, Δ*R***^***2***^ **= .087, *F*(1, 41) = 4.60, *p* = .038**			
Mean postnatal depression	-.161	2.136	-.805	.426
Mean postnatal anxiety	-.205	1.617	-1.017	.315
Maternal responsiveness	.311	10.236	2.145	**.038**

### Moderation effects of depression and anxiety scores on the relation between responsiveness and vocabulary

The regression model reported above tested whether depression and anxiety scores and maternal responsiveness ratings accounted for variance in vocabulary scores. When all three independent variables were entered into the model, maternal responsiveness was the only significant predictor of vocabulary size. This pattern could indicate that maternal depression and anxiety may be acting as moderators of the relation between maternal responsiveness and infants’ vocabulary size. To assess this possibility directly, we constructed two moderation models with maternal responsiveness as the predictor variable (X), vocabulary scores as the outcome variable (Y), and mean depression and anxiety scores as moderating variables (M) in Models 1 and 2 respectively.

The moderation analyses were conducted following the regression-based Conditional Process Analysis approach using the custom dialog “PROCESS” (Version 3.3) [74] in SPSS. This approach adds a second step to the hierarchical regression model described above and includes an additional test of the interaction between M and X to determine whether the interaction is a significant predictor of variance in Y (see [74] for further details). In this second step the standard errors and 95% confidence interval (CI) of the moderation effect are bootstrapped and bias-corrected (based on 5000 samples). To assist with its interpretation and to avoid multicollinearity, the analysis package automatically standardises all variables by centering them around the Mean, i.e., Mean and +/- 1 SD from the Mean.

The first moderation model was conducted to examine the possibility that mothers’ anxiety symptoms (M) moderate the relation between maternal responsiveness (X) and infant vocabulary scores (Y). In the first step, two variables were included: maternal responsiveness and anxiety. These two variables accounted for a significant amount of variance in infant vocabulary, *Adj R2* = .170, *F*(1, 42) = 5.519, *p* = .007. The maternal responsiveness and anxiety variables were used to create the maternal responsiveness × anxiety interaction term. This interaction term was added to the model in Step 2, which was not statistically significant, Δ*R2* = .057, *F* change (1, 41) = 3.167, *p* = .083. Maternal anxiety symptoms did not, therefore, have a significant moderating effect on the relation between responsiveness and vocabulary.

A second moderation model was conducted to examine the possibility that mothers’ depression symptoms (M) moderate the relation between maternal responsiveness (X) and infant vocabulary scores (Y). In the first step, two variables were included: maternal responsiveness and depression. These two variables accounted for 16% of variance in infant vocabulary, *Adj R2* = .16, *F*(2, 42) = 5.27, *p* = .009. The interaction term of maternal responsiveness × depression was added to the model in Step 2. This accounted for 9% of variance in infants’ vocabulary scores, Δ*R2* = .093, *F* (3, 41) = 5.391, *p* = .025. Maternal depression symptoms, therefore, had a moderating effect on the relation between maternal responsiveness and vocabulary.

**Fig 1:**
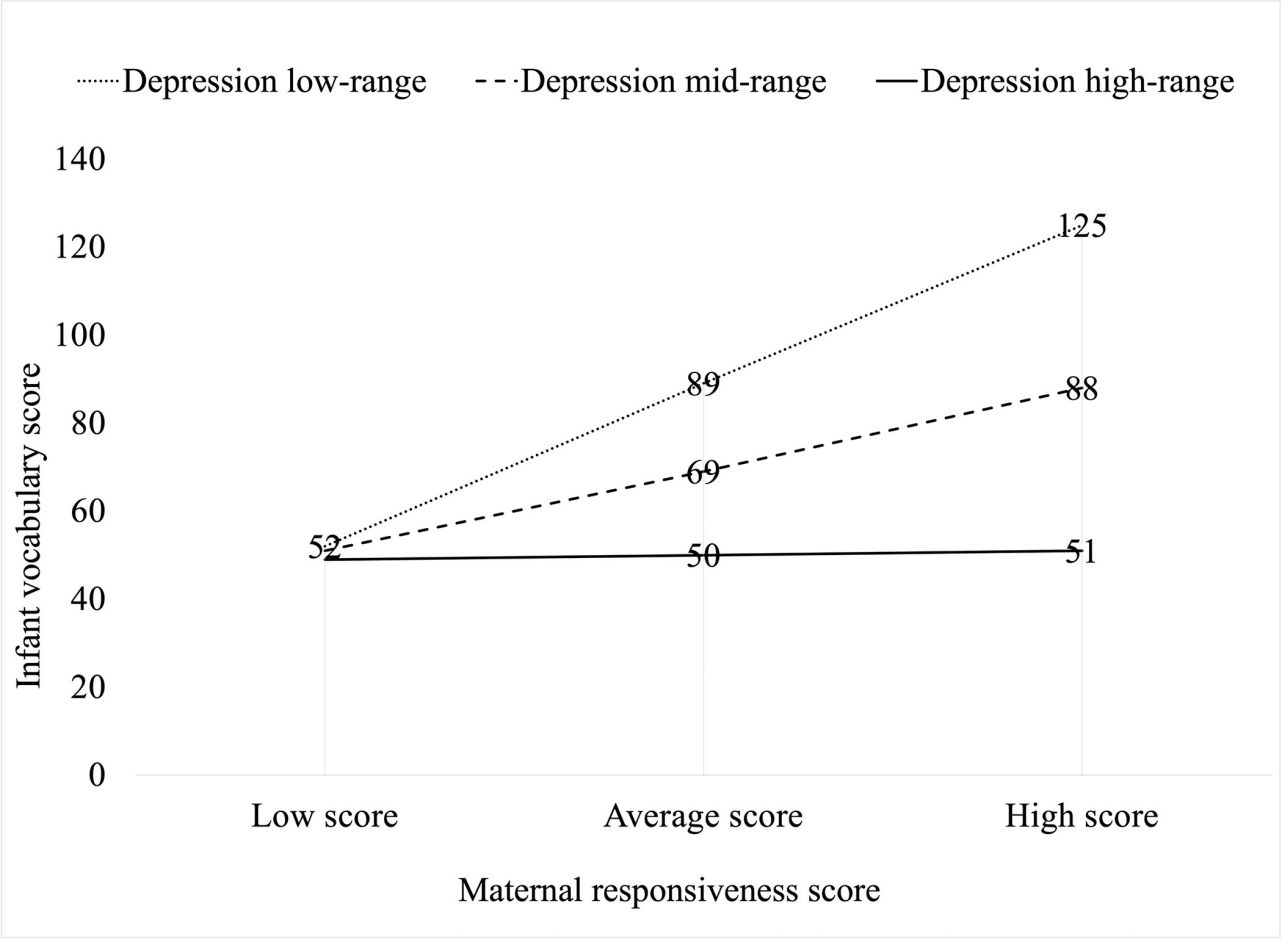
An interaction plot illustrating the moderation effect of maternal depression on the relation between maternal responsiveness and infant vocabulary scores.

To assist with the interpretation of the nature of the moderation effect of depression symptoms on the relation between maternal responsiveness and infants’ vocabulary size, interaction points were plotted in Fig 1 using model-generated weighted scores representative of three categories (low range, mid-range, and high range) for both maternal responsiveness and depression variables. As can be seen, maternal depression symptoms had a *decreasing* influence on the relation between maternal responsiveness and infants’ vocabulary scores, but this influence was conditional. For mothers with depression scores in the low or mid-range, maternal responsiveness was positively related with infants’ vocabulary size. However, for mothers with depression scores in the high range, this relation was not present. Regardless of their maternal responsiveness levels, infants’ vocabulary scores remained low.

## Discussion

This study confirms that maternal responsiveness observed in the first year of infants’ life relates to their language abilities developed in the second year. It demonstrates that this relation was moderated by the severity of mothers’ depression symptoms during the postnatal period. Consistent with our prediction, scores from a global rating scale of maternal responsiveness predicted expressive vocabulary size at 18-months over and above maternal depression and anxiety symptoms. Also, further examination revealed that the severity of mothers’ depression symptoms moderated this effect of maternal responsiveness on vocabulary size.

### Maternal responsiveness and vocabulary size

Maternal responsiveness was the only significant predictor of infants’ later vocabulary size in our regression model. This finding highlights the importance of the social context and qualitative aspects of infants’ language experience over and above maternal depression and anxiety symptoms. This result also supports language development theories that emphasise the role of social interactions, and the social constructivist view that supports a relation between social interactions and language acquisition [75, 76]. In fact, maternal depression and anxiety scores were not significant predictors of infant vocabulary size in our regression model, suggesting that on their own they do not explain individual differences in language development. While this finding does not align with our prediction and some previous studies [34, 35], it is consistent with research suggesting that additional maternal factors such as the quantity of speech input influence infant vocabulary size. For example, Brookman and colleagues [48] reported that infants of mothers with depression and anxiety symptoms were exposed to fewer numbers of conversational turns and produced fewer vocalisations compared to infants of mothers with no emotional health concerns. These social aspects of language in turn predicted vocabulary size at 18-months of age. In broad terms, the link between maternal responsiveness and vocabulary size reported here highlights the importance of the qualitative aspects of infants’ language experience in the first year of life (e.g., maternal responsiveness).

Contingency is a key component of maternal responsiveness, which refers to a mother’s response being conceptually and temporally linked to changes in her infant’s behaviour [77]. Infants strongly rely on time windows to associate words with objects [78] as their linguistic knowledge and associative networks are in a formative stage. When mothers’ vocal responses are temporally contingent (time-locked) to their infants’ vocalisations, both the quality and quantity of infants’ vocalisations increases [23, 24]. While this was not specifically examined in the present study, disruptions to contingency might explain the positive relations between maternal responsiveness and expressive vocabulary. It has also been posited that maternal responsiveness not only predicts, but also promotes language development [3]. Further, the association between maternal responsiveness and infant vocabulary size reported here is consistent with previous reports of similar links with pre-schoolers’ expressive and receptive language skills [21], and expressive language milestone achievements [27]. Further, the relation between maternal responsiveness and infant vocabulary in the first year of life may reflect the stability in maternal responsiveness during this period [79]. It certainly supports the important role that mothers have in maintaining and repairing any disruptions during the flow of conversational exchanges with their infants [80].

In addition to contingency, there are other mechanisms by which maternal responsiveness is proposed to promote infants’ language abilities, and that could account for the relation between maternal responsiveness and infant vocabulary size observed in this study. For example, responsiveness fosters infants’ secondary intersubjectivity by reinforcing the social and communicative functions of language, which may indirectly facilitate language development [81]. As a mother provides appropriate responses that are attuned to her infant’s communication cues, emotions, interests, and intentions, she reinforces the intersubjective nature of experiences, which illuminates her role as an interpreter of a shared world [27]. Further, in following the child’s lead, a mother also provides optimum occasions for language learning through focusing speech on what the child is attending to and by providing labels for objects and events under joint attention [82]. Infants also play an important role during interactions, which is why maternal input that is engaging and responsive may be particularly helpful in supporting language acquisition [83].

### Depression (not anxiety) symptoms moderate the effect of maternal responsiveness on vocabulary size

Our results are consistent with previous research linking maternal depression and infant language development to disruptions in mother–infant interactions [14, 46]. However, our findings extend the existing literature by suggesting that the relation between maternal responsiveness and depression and anxiety symptoms in the first year of life is neither guaranteed nor always linear. The level of maternal responsiveness in our sample of mother–infant dyads was not correlated with mothers’ depression and anxiety scores. If our analysis stopped at this point, it could appear that these important constructs were not related to each other despite empirical and theoretical literature pointing to the contrary [84, 85]. Here, we demonstrate for the first time that this is as a *moderation* relation. That is, depression symptoms reduce the positive influence that maternal responsiveness has on infants’ vocabulary. A novel finding is that depression symptoms, when mild to moderate, reduce (i.e., moderate) the effect of maternal responsiveness on vocabulary size. When depression symptoms were high, there was a negative impact on infants’ vocabulary size regardless of different levels of maternal responsiveness. This suggests that interventions that focus on maternal responsiveness should not be considered as a stand-alone treatment for mothers with higher depressive symptoms, which could result in little impact on their child’s language acquisition. It also suggests that interventions designed to reduce the severity of maternal depression symptoms (e.g., psychological counselling and psychopharmacology) are important in supporting infants’ vocabulary development.

Due to the interaction between depression symptoms and maternal responsiveness levels in regard to their impact on infant vocabulary, it would seem pertinent to consider both depression *and* responsiveness together when screening mother–infant dyads for developmental risk in the postnatal period. In Western societies, it is not uncommon for community services to use self-reported depression and psychosocial tools to screen for early signs of risk for adverse developmental outcomes for infants. Findings from the present study confirm that screening for severity of depression is important given that it functions as a moderator on the relation between maternal responsiveness and vocabulary. It also shows that screening mother–infant dyads for depression in isolation from maternal responsiveness will not provide a complete picture of the potential risks to infants’ developmental trajectory. From the visual representation of the moderation effect in Fig 1, it is clear that both, the level of depression *and* the level of maternal responsiveness matter with regard to vocabulary size growth, i.e. both have a negative impact on vocabulary size, but the interaction of depression symptoms *and* responsiveness levels together explains additional variance in vocabulary size. The moderation analysis supports the importance of screening for maternal responsiveness in the postnatal period in addition to assessments of maternal emotional health.

These results have the potential to inform research-based screening techniques for the early identification of infants at risk for language delay and provide direction for the design and implementation of early psychological interventions with mother–infant dyads at critical time points in infants’ development [86, 87]. For example, previous research studies suggest that while medication and psychological treatments can assist in the reduction of maternal depression symptoms [88, 89], they do not necessarily improve the quality of the mother–infant interaction and mitigate the risks to child development [42, 90, 91]. While the quantity of maternal interactions with their infant plays a role, the *quality* of maternal responsiveness (as captured by the PaRRiS Parental Responsiveness Scale) is also important in influences early language abilities [85]. Both screening and intervention strategies must therefore consider the levels of maternal responsiveness in addition to maternal depression scores in order to address all levels of inferred risk. As can be seen from the interaction plot in Fig 1, a mother with low maternal responsiveness may still have an infant who is at-risk for language delay regardless of her level of depression symptoms. Treating these mothers for depression without considering the *quality* of the interactions with their infant, that is maternal responsiveness, is unlikely to mitigate the risk of language delay for these infants. Furthermore, interventions that focus on increasing maternal responsiveness in mothers with low to moderate levels of depression increase the likelihood of gains in infant language acquisition. On the other hand, to promote language acquisition in infants of mothers with high depressive symptoms, interventions need to focus on both improving a mother’s mood in addition to her responsiveness to her infant; these are also likely to exist in a two-way relation where a mother’s improved mood may increase her responsiveness to her child and vice versa.

Our finding that maternal anxiety symptoms were not associated with and did not interact with maternal responsiveness in relation to vocabulary size, is congruent with previous findings establishing an inconsistent relationship between maternal anxiety and language development [92]. For example, there is evidence that mothers with high anxiety are less responsive and engaged with their infants [14, 46]. In contrast, we find no difference in maternal sensitivity between anxious and non-anxious mothers [46, 89]. Studies examining the association between maternal anxiety and adverse child developmental outcomes are also inconsistent. Some studies have found that infants of mothers with anxiety show poorer performance on cognitive tasks compared with infants of non-anxious mothers (see [43]). Other studies have not found a link between maternal anxiety and infants’ cognitive abilities (e.g., [93]. The inconsistent findings noted above may be due to the high co-morbidity between depression and anxiety and the limited homogeneity in both defining and measuring anxiety [38]. These factors may have contributed to the findings reported here.

There are several limitations that need to be considered with the findings reported in this study. First, even though we recruited a community sample, we did not achieve representation of lower SES families, which is a goal for future research that should also aim to recruit larger samples to improve the generalisability of findings. Second, vocabulary size was measured using a parental report rather than an objective measure. The validity of this method is well established (see [71] for the specific adaptation of the CDI used here), but the effects of maternal depression and anxiety on this type of parental report are unknown. For instance, it has been previously shown that mothers with depression are less sensitive to their children’s communicative cues [13, 46], so mothers with more severe symptoms could underestimate their child’s vocabulary size. This is a possibility, but we note that a previous study with a sub-sample of children included here demonstrated that children of depressed mothers underperformed children of non-depressed mothers in an objective experimental measure of lexical ability, which was significantly correlated with parental reports of vocabulary size [36]. Therefore, it is unlikely that low vocabulary scores observed for infants of mothers with more severe depression and anxiety symptoms were due to maternal reporting biases, but rather they were indicative of individual differences in lexical abilities in this sample.

An advantage of a continuous analytic approach is that it enabled us to consider subclinical levels of depression and anxiety, which are associated with functional and social impairment [64]. Findings suggest that higher levels of subclinical depression moderate down the benefits of maternal responsiveness, highlighting the importance of managing depression symptoms early before they reach clinical thresholds. Mothers with a history of depression, even when receiving treatment, should be more closely screened for symptoms. However, this recruitment approach may have impacted the findings reported here and may explain some inconsistencies with previous research. Existing literature suggests that both chronic maternal anxiety and/or depression are more likely to impact the quality of mother-infant interactions and children’s language development. For example, children of mothers with chronic depression have lower language scores when compared to children of mothers with less chronic depression [34]. Future research should seek to examine the impact on maternal responsiveness and vocabulary size using a clinical population.

Interestingly, our findings did not yield a significant relation between maternal responsiveness and depression or anxiety symptoms, which may be related to the examination of a non-clinical sample group as we discuss above. Also, a five-point scale used to assess maternal responsiveness narrows the range of variability in participants’ scores. It is therefore plausible that a more fine-grained and labour-intensive measurement may have been more sensitive to variability in maternal responsiveness that is associated with these mental health conditions. The PaRRiS measure of parental responsiveness measures parental input that is clearly related to child language outcomes and not as closely aligned with attachment-based theory of parent-child interactions. An association between maternal mental health and measures of parent-child interaction, may be more likely to occur when the parent-child interaction captures measures of the emotional bond, such as maternal warmth. Examining other dimensions of the quality of mother-infant interactions in addition to responsiveness may also assist in determining the extent to which the quality of maternal input is related to depression and anxiety symptoms and language abilities. However, administering a routine screening of maternal responsiveness in a clinical context, would require a tool that can be administered rapidly and in real-time, without the requirement of extensive training. Results from the present study support the utility of the PaRRiS as a potential screening tool in the postnatal period, by demonstrating its ability to detect variations in maternal responsiveness in the first year of life, that were also predictive of vocabulary size at 18-months. This extends previous research conducted with older children [55] by highlighting the PaRRiS as a potential screener for identifying mother–infant dyads at risk for developing small vocabulary sizes in the context of postnatal depression symptoms.

In conclusion, this study reveals that maternal responsiveness in the first year of life is a stronger predictor of infant vocabulary size than maternal depression or anxiety scores. This suggests that it is not depression and/or anxiety per se that lead to adverse outcomes in infants’ lexical abilities, but it is the potential impact of these symptoms on the social aspects of infants’ early language experiences. Most importantly, this study is the first to provide evidence for the moderation effect of depression symptoms on maternal responsiveness and infants’ vocabulary, whereby higher depression scores were observed to moderate the effect of maternal responsiveness on infant vocabulary size. It is therefore important to identify early subclinical symptoms of maternal depression and to treat these symptoms first, followed by providing mothers with the necessary support to foster maternal responsiveness during interactions with their infants. Mothers with higher depressive symptoms need more comprehensive support to assist them to reduce depression symptoms so that improvements in their responsiveness have the potential to be protective for their child’s language acquisition.

## Supporting information

S1 File Parental responsiveness rating scale. Parental Responsiveness Rating Scale.
(DOCX)

S1 Table Pearson correlation Tables. Pearson correlation analyses for individual CESD-R and STAI scores across the 6-, 9-, 12-, and 18-month time points.
(DOCX)

S2 Table Pearson correlations Table. Pearson correlations with maternal responsiveness, maternal age and education, infant expressive vocabulary, and mean depression and anxiety measures.
(DOCX)

S3 Table Multiple regression models with covariates. Multiple regression models with co-variates (maternal education and age), maternal emotional health, and maternal responsiveness predicting expressive vocabulary.
(DOCX)
