# Supplementary material for: Effects of maternal depression on maternal responsiveness and infants’ expressive language abilities
Source: PLoS One. 2023 Jan 11;18(1):e0277762. doi: 10.1371/journal.pone.0277762 (PMC9833548; doi:10.1371/journal.pone.0277762)
Supplement: S1 File — Parental Responsiveness Rating Scale. (DOCX) [file pone.0277762.s001.docx]

**Supplementary Material**

S2.

*Parental Responsiveness Rating Scale (PaRRiS) (Levickis et al., 2020)*

**Rating Definitions**

1 = very low

Parent rarely responds in a developmentally appropriate way either verbally or non-verbally to any of Child’s gestures or verbalisations AND Parent attempts to redirect Child’s behaviour, rather than following Child’s interests.

2 = low

Parent responds occasionally in a developmentally appropriate way either verbally or non-verbally to Child’s gestures or verbalisations AND/OR Parent spends more time attempting to redirect Child’s behaviour than following Child’s interest.

3 = moderate (average)

Parent spends some time responding in a developmentally appropriate way either verbally or non-verbally to Child’s gestures or verbalisations, and some time ignoring them AND/OR Parent spends equal time following Child’s interest and redirecting Child’s behaviour.

4 = high

Parent often responds in a developmentally appropriate way either verbally or non-verbally to Child’s gestures or verbalisations AND/OR Parent spends more time following Child’s interest than redirecting Child’s behaviour.

5 = very high

Parent frequently responds in a developmentally appropriate way either verbally or non-verbally to Child’s gestures or verbalisations AND Parent rarely attempts to redirect Child’s focus from the current activity but follows Child’s interests.

*Note:* Specification of extent of parental directiveness: ‘redirecting the child’s behaviour’ refers to redirecting the child’s attention away from their current play and interests at that point in time.
