# Supplementary material for: Effects of maternal depression on maternal responsiveness and infants’ expressive language abilities
Source: PLoS One. 2023 Jan 11;18(1):e0277762. doi: 10.1371/journal.pone.0277762 (PMC9833548; doi:10.1371/journal.pone.0277762)

**Supplementary Material**

S1. Tables

Pearson correlation analyses for individual CESD-R and STAI scores across the 6-, 9-, 12-, and 18-month time points. Moderate to high correlations observed for each instrument confirm the reliability and stability of these assessments within our sample.

1. *Centre for Epidemiologic Studies Depression Scale-Revised (CESD-R)*


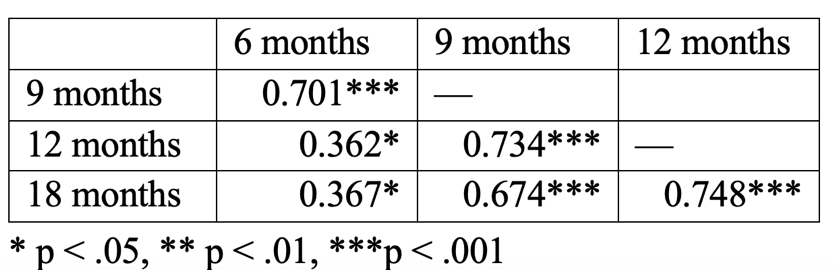


1. *State Scale of the State-Trait Anxiety Inventory (STAI)*


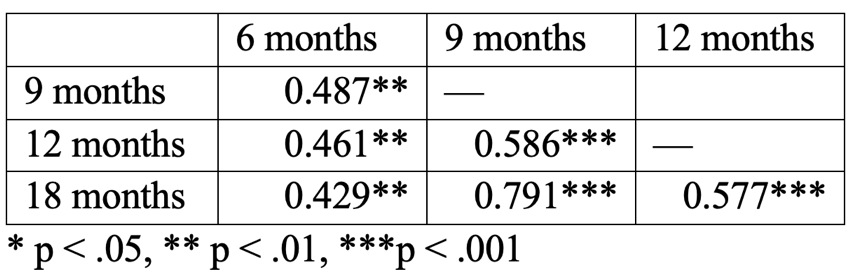

Supplement: S1 Table — Pearson correlation analyses for individual CESD-R and STAI scores across the 6-, 9-, 12-, and 18-month time points. (DOCX) [file pone.0277762.s002.docx]
