# Supplementary material for: Effects of maternal depression on maternal responsiveness and infants’ expressive language abilities
Source: PLoS One. 2023 Jan 11;18(1):e0277762. doi: 10.1371/journal.pone.0277762 (PMC9833548; doi:10.1371/journal.pone.0277762)
Supplement: S2 Table — Pearson correlations with maternal responsiveness, maternal age and education, infant expressive vocabulary, and mean depression and anxiety measures. (DOCX) [file pone.0277762.s003.docx]

**Supplementary Material**

S3.

*Pearson correlations with maternal responsiveness, maternal age and education, infant expressive vocabulary, and mean depression and anxiety measures.*

|  | 1. MR | 2. Maternal education | 3. Maternal age | 4. Ozi | 5. Mean  Depression |
| --- | --- | --- | --- | --- | --- |
| 1.MR | 1 |  |  |  |  |
| 2. Maternal education | .279 | 1 |  |  |  |
| 3. Maternal age | -.300* | .004 | 1 |  |  |
| 4. Ozi | .367* | .123 | -.64 | 1 |  |
| 5. Mean depression | .034 | .034 | -.151 | -.275 | 1 |
| 5. Mean anxiety | -.191 | .025 | .110 | -.363* | .708** |

*Note.* * *p* <. 05; ** *p* < .01; *** *p* < .001; OZI = Australian English Communicative Development Inventory; MR = Maternal responsiveness; Mean depression = average CESD-R scores; Mean anxiety = average STAI scores.
