# Supplementary material for: Effects of maternal depression on maternal responsiveness and infants’ expressive language abilities
Source: PLoS One. 2023 Jan 11;18(1):e0277762. doi: 10.1371/journal.pone.0277762 (PMC9833548; doi:10.1371/journal.pone.0277762)
Supplement: S3 Table — Multiple regression models with co-variates (maternal education and age), maternal emotional health, and maternal responsiveness predicting expressive vocabulary. (DOCX) [file pone.0277762.s004.docx]

**Supplementary Material**

S4.

*Multiple regression models with co-variates (maternal education and age), maternal emotional health, and maternal responsiveness predicting expressive vocabulary.*

| **Predictors** |  | | | | |
| --- | --- | --- | --- | --- | --- |
| **Removed covariates** | ***R^2^*= .14, *F* (3, 40) = 3.364, *p* = .028** | | | | |
|  | **B** | ***SEM*** | ***β*** | ***T*** | ***P*** |
| Maternal education | 2.644 | 7.712 | .052 | .345 | .732 |
| Maternal age | .532 | 2.207 | .038 | .241 | .811 |
| **Entered predictor variables** |  | | | | |
| Mean postnatal depression | -1.812 | 2.161 | -.171 | -.839 | .407 |
| Mean postnatal anxiety | -1.202 | 1.693 | -.149 | -.710 | .482 |
| Maternal responsiveness | 21.652 | 10.217 | .321 | 2.119 | .040 |
